# Delay in hip reductions due to the advent of rapid CT scans in the trauma setting

**DOI:** 10.1007/s00402-026-06192-9

**Published:** 2026-02-02

**Authors:** John Hwang, David Ahn, Caroline Preston, Michael S. Sirkin, Joseph D. Galloway, Mark C. Reilly, Mark R. Adams

**Affiliations:** 1 Department of Orthopaedics, Mount Carmel Health System, Grove City, OH USA; 2 Department of Orthopaedic Surgery, Rutgers New Jersey Medical School, Newark, NJ USA; 3 University of South Florida, Tampa, USA

**Keywords:** Hip dislocation, Trauma, Diagnostic imaging, Perioperative optimization

## Abstract

**Introduction:**

: With increasing reliance on computed tomography (CT) in trauma care, the use of anteroposterior (AP) pelvis radiographs has declined. This study examined whether omitting an initial AP pelvis film affected time to hip reduction and the need for additional CT imaging in patients with traumatic hip dislocations.

**Methods:**

We conducted a retrospective review at a Level I trauma center (2005–2016). Eligible patients were adults (> 17 years) with native hip dislocations evaluated under the Advanced Trauma Life Support (ATLS) protocol. Patients with incomplete records or irreducible hips requiring operative reduction were excluded. Data collected included patient demographics, AP pelvis use, CT imaging, time to reduction, and presence of acetabular or proximal femur fractures.

**Results:**

The study cohort consisted of 50 patients, 76% male (*n* = 38), with a mean age of 33 years (range, 18–68). High-energy motor vehicle accident or motorcycle crash accounted for 90% (*n* = 45) of injuries, and 94% (*n* = 47) were posterior dislocations. Associated fractures were present in 76% (*n* = 38). Patients were divided into those who had no AP pelvis radiograph prior to CT scan (N-APP group, *n* = 8; 16%) and those who obtained an initial AP pelvis radiograph on presentation (APP group, *n* = 42; 84%). All patients in the N-APP group received an additional CT pelvis scan, while none in the APP group did. Average time to reduction was significantly shorter in the APP group compared with the N-APP group (69 vs. 216 min, *p* < 0.05).

**Conclusions:**

Obtaining an initial AP pelvis radiograph provided a rapid and reliable means of diagnosing hip dislocations. Adherence to ATLS guidelines by performing a pelvic film before CT shortened time to reduction and prevented unnecessary repeat CT imaging in adult patients with traumatic native hip dislocations.

## Introduction

Traumatic dislocations of the hip often occur following high-energy trauma and require emergent care. These injuries are most seen in the setting of motor vehicle accidents [1]. Prognosis is variable, but obtaining a closed reduction as quickly as possible is ideal to decrease the chance of avascular necrosis of the femoral head and minimize the incidence of post-traumatic arthritis of the hip [2]. Historically, a dislocated hip is diagnosed on an AP pelvis x-ray performed as part of the Advanced Trauma Life Support (ATLS) protocol, with the ATLS algorithm mandating a pelvic radiograph during the initial assessment of the patient [3]. However, there is an increasing contingent of practitioners questioning the utility of plain film pelvic radiographs in the era of the readily available Computerized Tomography (CT) [4]. Across several trauma systems, CT-first workflows have been adopted for hemodynamically stable blunt-trauma patients, with some protocols omitting the screening AP pelvis film to expedite transport to CT. Reports from surgery, radiology, and critical care communities describe reduced use of routine pelvic radiographs and support streamlined initial imaging through extended Focused Assessment with Sonography for Trauma (eFAST) and early whole-body CT in stable patients [4–9].

Current orthopaedic guidelines require a CT scan after reduction of a dislocated hip to evaluate for concentric reduction and intra-articular loose bodies [10, 11]. In the situation of a hemodynamically stable patient without an obvious physical exam finding, the general surgery trauma literature advocates forgoing the initial AP pelvis to obtain the CT scan more quickly [12]. For these reasons, it has become a more common occurrence for the trauma CT to be performed before an AP pelvis has been obtained. In the setting of a dislocated hip, foregoing the traditional ATLS protocol (i.e., AP pelvis radiograph), often results in the diagnosis being made on the CT scan. This then results in a delay in reduction, followed by a second CT scan to evaluate the joint. In cases where two CT scans are performed, patients may incur additional radiation exposure, with a typical pelvis CT delivering approximately 3–8 mSv, and they may face increased healthcare resource utilization. When feasible, avoiding an additional CT scan aligns with principles of radiation safety and cost-conscious care [13−6].

The clinical presentation of a patient with a hip dislocation is typically characteristic and should prompt an immediate AP pelvis film. The purpose of this study was to show that a substantial number of patients with hip dislocations did not receive an AP pelvis film prior to CT. We hypothesized that for patients with traumatic hip dislocations, a significant number were initially diagnosed on CT scan. Secondarily, we hypothesized that by not performing an initial AP pelvic radiograph prior to a CT scan, these patients would require additional CT scans and have an increased time to hip reduction.

## Methods

Institutional review board approval was obtained prior to accessing the trauma database for the collection of patient information. Patients were searched for and selected from our institution’s trauma database by ICD-9 and ICD-10 diagnosis codes for hip dislocation during the years 2005–2016. Inclusion criteria consisted of patients over the age of 17, initial evaluation with ATLS protocol (per hospital records), and native hip dislocation. Patients with incomplete medical records and irreducible hips that required reduction in the operating room were excluded from the study. Sixty-two patients were identified, and twenty-two patients were excluded because of incomplete medical records, irreducible hips in the emergency department, patients with prior hip arthroplasty, and pediatric patients.

Patient demographics, including age and gender, were recorded. In addition, laterality of dislocation, mechanism of injury, and presence of associated fracture were identified. Next, a chart review was performed to assess whether the patient had an AP pelvis film prior to CT and to determine the total number of CT pelvis scans each patient obtained on initial evaluation. The patients who did not obtain an AP pelvis radiograph prior to the CT scan were grouped in the No AP Pelvis (N-APP) group, and the patients who obtained AP pelvis radiographs prior to a CT scan were grouped in the AP Pelvis (APP) group. Time from presentation until radiographic confirmation of hip reduction was also recorded.

Due to the retrospective nature of this review and inconsistent documentation in the medical record, detailed data regarding pain scores, analgesic use, operative timing, or neurologic findings such as sciatic nerve palsy were not reliably available and therefore were not collected. Similarly, institutional billing and cost data were not consistently documented and were not included in the analysis. These factors are acknowledged as limitations of the dataset.

Chi-squared analysis was utilized to compare the prevalence of additional CT scans performed in the N-APP group as compared to those in the APP group. Furthermore, a one-tailed T-test was used to determine whether a significant delay in time to radiographic confirmation of hip reduction between the two groups. A P value of < 0.05 was selected for the determination of statistical significance.

## Results

There were 50 patients who sustained hip dislocations from 2005 to 2016 who met our inclusion and exclusion criteria. The group was predominantly male at 76%. Patient age ranged from 18 to 68, with an average age of 33. The predominant mechanism of injury was a high-energy motor vehicle accident or motorcycle crash, which together accounted for 90% of cases. The remaining cases were comprised of individuals who had fallen from heights or pedestrians who had been struck by motor vehicles. 55% had an associated acetabular fracture. Three patients had anterior dislocations, while the remaining patients had posterior dislocations.

There were eight patients (19%) who had CT scans while still dislocated without an initial AP pelvis film and who were grouped in the N-APP group. Of those eight patients in the N-APP group, 100% received an additional CT pelvis scan, while no patients in the APP group required additional CT pelvis scans (*P* < 0.05) (Table 1).

**Table 1:** Patient demographics, injury characteristics, and CT utilization by group (APP vs. N-APP)

	*N*-APP (8)	APP (42)	Total (50)	*p*-value
Gender				
Male	88% (7)	74% (31)	76% (38)	0.239
Female	12% (1)	26% (11)	24% (12)	
Laterality				
Right	88% (7)	67% (28)	70%(35)	0.406
Left	12% (1)	33% (14)	30% (15)	
Associated Fracture				
Yes	88% (7)	74% (31)	76% (38)	0.239
No	12% (1)	26% (11)	24% (12)	
Number of Pelvis CT				
1	0% (0)	100% (42)	84% (42)	**0.001**
2	100% (8)	0% (0)	16% (8)	

**Fig. 1:**
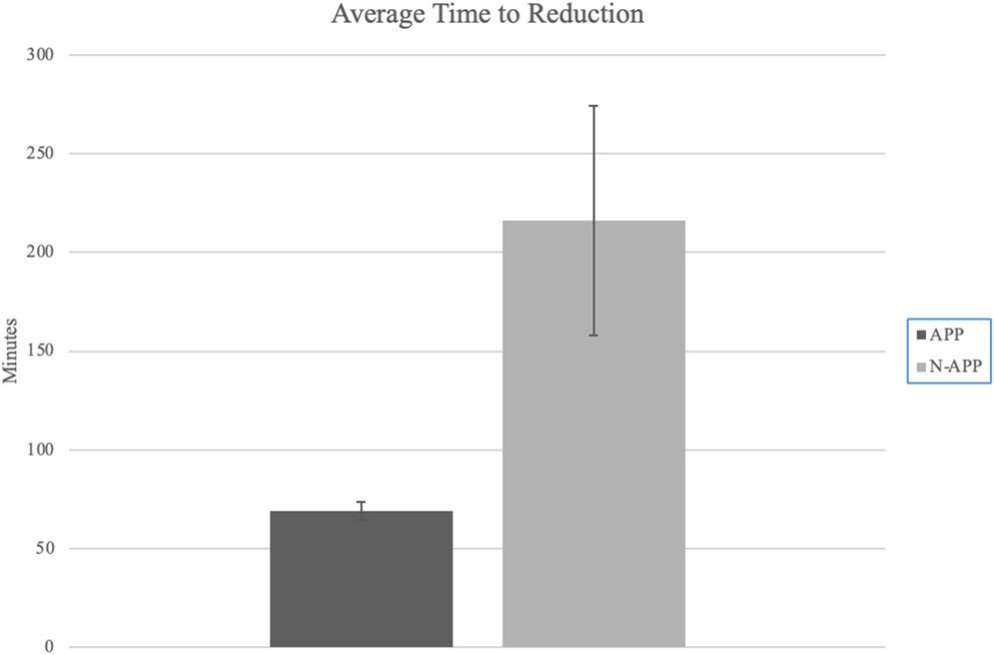
Average time to reduction for patients with traumatic hip dislocations comparing those who underwent an initial AP pelvis radiograph (APP group) versus those who did not (N-APP group). Error bars represent standard error of the mean. The difference between groups was statistically significant (*p* < 0.05)

The overall average time from presentation to the hospital to radiographic confirmation of hip reduction was 93 min. In the APP group average reduction time was 69 min (Standard Error − 4.8), while in the N-APP group, it was 216 min (Standard Error − 58.1), respectively (*p* = 0.039) (Fig. 1). In those that did not obtain an initial AP pelvic radiograph, there was an average of 2 h and 27 min (216 min vs. 69 min) delay in time to reduction (*p* < 0.05).

## Discussion

In this single-center retrospective review of traumatic hip dislocations, patients who did not receive an initial AP pelvis radiograph experienced substantially longer times to reduction and uniformly received a second CT scan. In contrast, patients who underwent AP pelvis radiography on presentation had significantly shorter reduction times and did not require additional CT imaging. These findings support continued adherence to ATLS principles by obtaining a screening AP pelvis radiograph in the initial trauma evaluation of suspected hip dislocation.

Traumatic hip dislocation and fracture-dislocation occur following high-energy trauma, typically seen with motor vehicle accidents [1]. These injuries often occur following high-energy trauma, typically seen with motor vehicle accidents [1]. Due to the severity of these traumas, these patients present to the emergency department with other concomitant injuries that require ATLS management [13–15]. The traditional ATLS algorithm includes obtaining an emergent AP pelvis during the evaluation of these patients [3]. Recent studies in trauma and emergency medicine fields have suggested that the use of initial AP pelvis radiograph may be unnecessary with the advent and accessibility of CT imaging [4, 12, 16]. Conversely, orthopaedic literature continues to recommend a pelvis radiograph during initial management of the high-energy traumatized patients [17, 18].

Urgent reduction of the hip dislocation continues to be recommended due to the high risk of avascular necrosis associated with this injury. In adults, the cervical arteries, which branch from the medial femoral circumflex artery, provide the main blood supply to the femoral head. Injury to these vessels could lead to avascular necrosis of the femoral head. With posterior dislocation, kinking or injury could occur to these vessels. A cadaveric study by Yue et al. examined six hips after they were forcefully dislocated posteriorly. The research found that there were filling defects in the circumflex arteries following dislocation [19].

A study performed in 1962 by Brav et al. found that 22% of patients who underwent reductions within 12 h, as opposed to 52% of patients whose reductions were delayed greater than 12 h, developed osteonecrosis [20]. A more recent retrospective study by Hougaard et al. found that, at the 5-year mark, 4% of those patients who underwent closed reduction within 6 h, as opposed to 58% of patients whose reductions were delayed greater than 6 h, developed osteonecrosis.

In our study, approximately 1 in 5 patients (19%) with a hip dislocation did not have plain pelvis radiographs on initial evaluation, resulting not only in a delay in hip reduction but also an increased exposure to radiation. When comparing average times to hip reduction for the two groups, the N-APP group was found to have an increase of greater than two hours in average time to reduction when compared to that of the APP group. As these times were based on initial arrival to the emergency department, the actual time from injury to reduction would be greater than our current presented time, as we did not incorporate the time accrued prior to the patient arriving in the emergency department. In the N-APP group, time from emergency department arrival to reduction ranged from 91 to 584 min (mean 216 min), with one patient exceeding 6 h and three patients exceeding 4 h. In contrast, the APP group demonstrated a range of 12 to 151 min (mean 69 min), with no patients exceeding 6 h. When considering additional pre-hospital time, it is likely that a greater proportion of N-APP patients surpassed the 6-hour clinical threshold cited in prior literature.

Another complication associated with traumatic hip dislocations is post-traumatic arthritis. Post-traumatic arthritis is the most common complication, with rates as high as 48% [20–22]. Prolonged time to reduction can increase the risk of injury to the femoral head and the articular surface. Movement of patients during transportation to CT imaging prior to hip reduction, especially in those patients with unknown dislocations, can cause continued injury to the articular cartilage and further increase the risk of injury to the femoral head.

While this is an area that is not yet fully understood, various studies have implied that increased radiation exposure poses a risk for radiation-induced carcinogenesis [23–25]. Currently, orthopaedic literature highly recommends CT scans following closed reduction of hip dislocations in order to evaluate for concentric reduction, loose bodies in the joint, and occult fractures [10, 11, 26]. Diagnosing and reducing hip dislocation during initial trauma management would prevent unnecessary radiation exposure, as the CT chest/abdomen/pelvis would be performed after the reduction. Our study found that 0% of those in the APP required additional CT scans, while 100% of those in the N-APP group required additional CT imaging. This additional CT scan results in increased radiation exposure. In addition to delays in reduction, repeat CT scanning increases cumulative radiation dose. A typical pelvis CT exposes patients to approximately 3–8 mSv, depending on scanner protocol and patient characteristics, contributing meaningfully to cumulative ionizing radiation exposure [27–30]. Although a post-reduction CT scan remains standard to confirm concentric reduction and exclude intra-articular fragments, performing two CT scans during initial trauma evaluation represents avoidable additional radiation when diagnosis can be established with an AP pelvis radiograph. Furthermore, repeated CT imaging increases resource utilization and patient charges, reinforcing the value of an initial screening radiograph in trauma workflows.

Our study demonstrates several limitations. First, although prior literature has established the consequences of delaying hip reductions, our study did not determine the clinical sequela of the delay in diagnosis within our population. Detailed documentation of pain scores, analgesic use, neurologic findings such as sciatic nerve palsy, and hospital billing data was inconsistent in the medical record, which precluded reliable analysis of these variables. Furthermore, the retrospective nature of our study did not allow us to consider possible other factors associated with the delay in reduction. Finally, this study was limited to one institution with a limited sample size. A larger data set would help strengthen the significance of our findings.

## Conclusion

During initial management of traumatized patients, pelvis plain radiographs continue to be recommended for evaluation of hip dislocations. Our study demonstrated that patients with traumatic hip dislocations who received a plain radiograph of the pelvis during initial trauma evaluation had decreased time to reduction and fewer CT scans.

## Data Availability

No datasets were generated or analysed during the current study.
